# Rhythm & Resilience: Drumming as a Pathway to Wellness in Later Life

**DOI:** 10.1093/geroni/igaf122.710

**Published:** 2025-12-31

**Authors:** Emily Ihara, Michelle Hand

**Affiliations:** George Mason University, Fairfax, Virginia, United States; George Mason University, Fairfax, Virginia, United States

## Abstract

Music engagement in later life fosters cognitive, emotional, and social well-being. Drumming, a uniquely accessible and embodied musical activity, holds promise as a non-pharmacological intervention for older adults, including those living with intellectual and developmental disabilities (IDD) and neurodegenerative conditions. This presentation highlights findings from Rhythm & Nature, a drumming and horticulture-based intervention designed to promote wellness and engagement among older adults with IDD. Drumming provides a multisensory experience, activating broad neural networks and facilitating bilateral stimulation, which is theorized to enhance cognitive processing, emotional regulation, and motor coordination. Group drumming fosters interpersonal synchrony, reinforcing social connections and reducing feelings of isolation. Participants in Rhythm & Nature reported increased confidence, improved mood, and eagerness for future sessions. Additionally, drumming’s adaptability enables individuals with diverse abilities to participate, reinforcing agency and self-expression. This session will explore the multidimensional benefits of drumming in older populations, emphasizing its impact on cognitive health, emotional resilience, and social inclusion. Findings will be discussed within a broader framework of music-based interventions for older adults, underscoring drumming’s potential to support intergenerational engagement and community-building. In reimagining music for older adults, we propose that drumming – rooted in rhythm and collective participation – offers a bold and innovative avenue for enhancing well-being in later life.

